# Targeting a free viral fraction enhances the early alert potential of wastewater surveillance for SARS-CoV-2: a methods comparison spanning the transition between delta and omicron variants in a large urban center

**DOI:** 10.3389/fpubh.2023.1140441

**Published:** 2023-07-20

**Authors:** Liang Zhao, Qiudi Geng, Ryland Corchis-Scott, Robert Michael McKay, John Norton, Irene Xagoraraki

**Affiliations:** ^1^Department of Civil and Environmental Engineering, Michigan State University, East Lansing, MI, United States; ^2^Great Lakes Institute for Environmental Research, University of Windsor, Windsor, ON, Canada; ^3^Great Lakes Center for Fresh Waters and Human Health, Bowling Green State University, Bowling Green, OH, United States; ^4^Great Lakes Water Authority, Detroit, MI, United States

**Keywords:** wastewater surveillance, SARS-CoV-2, COVID-19, virus adsorption-elution, polyethylene glycol precipitation, filtration, lead/lag time, dynamic time warping

## Abstract

**Introduction:**

Wastewater surveillance has proven to be a valuable approach to monitoring the spread of SARS-CoV-2, the virus that causes Coronavirus disease 2019 (COVID-19). Recognizing the benefits of wastewater surveillance as a tool to support public health in tracking SARS-CoV-2 and other respiratory pathogens, numerous wastewater virus sampling and concentration methods have been tested for appropriate applications as well as their significance for actionability by public health practices.

**Methods:**

Here, we present a 34-week long wastewater surveillance study that covers nearly 4 million residents of the Detroit (MI, United States) metropolitan area. Three primary concentration methods were compared with respect to recovery of SARS-CoV-2 from wastewater: Virus Adsorption-Elution (VIRADEL), polyethylene glycol precipitation (PEG), and polysulfone (PES) filtration. Wastewater viral concentrations were normalized using various parameters (flow rate, population, total suspended solids) to account for variations in flow. Three analytical approaches were implemented to compare wastewater viral concentrations across the three primary concentration methods to COVID-19 clinical data for both normalized and non-normalized data: Pearson and Spearman correlations, Dynamic Time Warping (DTW), and Time Lagged Cross Correlation (TLCC) and peak synchrony.

**Results:**

It was found that VIRADEL, which captures free and suspended virus from supernatant wastewater, was a leading indicator of COVID-19 cases within the region, whereas PEG and PES filtration, which target particle-associated virus, each lagged behind the early alert potential of VIRADEL. PEG and PES methods may potentially capture previously shed and accumulated SARS-CoV-2 resuspended from sediments in the interceptors.

**Discussion:**

These results indicate that the VIRADEL method can be used to enhance the early-warning potential of wastewater surveillance applications although drawbacks include the need to process large volumes of wastewater to concentrate sufficiently free and suspended virus for detection. While lagging the VIRADEL method for early-alert potential, both PEG and PES filtration can be used for routine COVID-19 wastewater monitoring since they allow a large number of samples to be processed concurrently while being more cost-effective and with rapid turn-around yielding results same day as collection.

## 1. Introduction

Wastewater surveillance has been widely adopted by researchers and health agencies as an effective tool for tracking Severe Acute Respiratory Syndrome Coronavirus 2 (SARS-CoV-2) in wastewater amid the Coronavirus Disease 2019 (COVID-19) pandemic (1–13). SARS-CoV-2 was first identified in Wuhan, Hubei, China, and was designated a Public Health Emergency of International Concern on January 30th, 2020, by the World Health Organization (WHO). COVID-19 was later declared a pandemic on March 11th, 2020 (who.int). Numerous studies have demonstrated that SARS-CoV-2 can be shed from the gastrointestinal tract of infected individuals and its viral RNA can persist and be detected in wastewater (14–18). To increase the sensitivity of the assay used to detect viral RNA in wastewater, samples are routinely concentrated prior to quantification (19–21).

Methods used in published studies to recover and concentrate SARS-CoV-2 viral RNA from wastewater encompass a wide range of techniques including Virus Adsorption-Elution (VIRADEL), polyethylene glycol precipitation (PEG), ultrafiltration, ultracentrifugation, concentrating pipette, filtration and so forth. Some of the methods, such as VIRADEL, exclude large solids and focus on free and suspended viral particles in supernatant wastewater. Other methods, such as PEG precipitation and filtration, target particulate matter and the associated viruses that are sorbed onto solids. Notably, this fraction may preferentially settle within the sewer when flow is reduced and likewise is susceptible to resuspension when flows are elevated (3, 22).

The recovery efficiencies of concentration methods are variable, differing between method, virus type and conditioning of the wastewater sample. Notably, VIRADEL was found to be effective for concentrating viruses from water samples with recovery efficiencies of more than 90% for poliovirus (23, 24), 54.4% for murine norovirus (MNV) (25), 51% for echovirus (26), 35% for enteric virus (27), and 4.7% for adenovirus (28). Likewise, PEG was found to be effective for concentrating viruses in water samples, with recovery efficiencies of 89.5% for echovirus (29), 86% for hepatitis A virus (30), 68% for poliovirus (30), and 56.7% (31) and 26.4% (32) for SARS-CoV-2. Filtration was reported to recover virus from wastewater samples with recovery efficiencies ranging from 26.7 to 65.7% for murine hepatitis virus (33), and 90% for human betacoronavirus OC43 (34).

Applying different concentration methods can achieve different goals. For instance, use of VIRADEL to concentrate SARS-CoV-2 can provide early warnings of impending COVID-19 cases (1, 3, 13). PEG precipitation is an economical and widely adopted method that allows a large number of samples to be processed concurrently and it is suitable for routine COVID-19 wastewater monitoring (22, 35). Likewise, filtration presents a cost-effective and simple approach commonly applied to recover cells and viral particles from environmental samples for nucleic acid extraction (36), which has also been applied to recovery of SARS-CoV-2 from wastewater (12, 35, 37, 38).

Here we present a comparison of three primary concentration methods (VIRADEL, PEG and filtration) to detect SARS-CoV-2 viral RNA in wastewater, in relation to COVID-19 cases amid the transition from Delta to Omicron Variants of Concerns (VOCs) circulating in the Detroit, MI metropolitan area. Similarities and correlations were examined among the three concentration methods with both normalized and non-normalized data. The lead/lag time of each method in relation to the total COVID-19 cases was also assessed. The results presented in this study will assist researchers and public health practitioners to select appropriate primary concentration methods for the recovery of SARS-CoV-2 from wastewater for different wastewater surveillance practices.

## 2. Materials and methods

**Figure 1:**
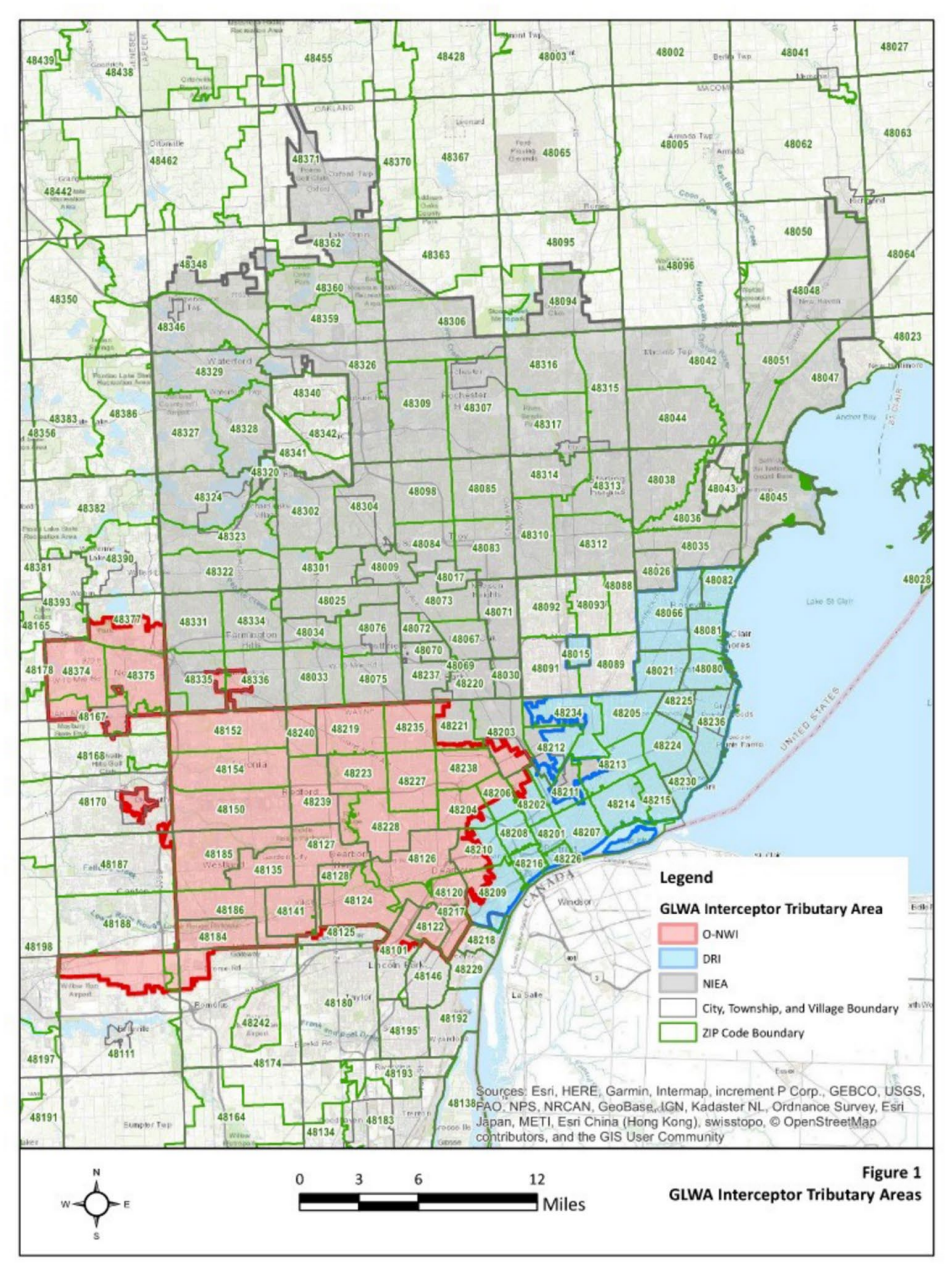
GLWA interceptor tributary map.

Untreated wastewater samples were collected weekly from the Water Resource Recovery Facility (WRRF) of the Great Lakes Water Authority (GLWA) located in Detroit, MI, United States, between October 1, 2021, and May 31, 2022. The WRRF serves the needs of Detroit and 76 area communities with a service area of more than 2,450 square kilometers serving nearly 4 million people. WRRF collects and treats stormwater, as well as residential, industrial, and commercial waste, depending on service areas, with its semi-combined sewershed system. WRRF receives wastewater via three main interceptors including the Detroit River Interceptor (DRI), the North Interceptor-East Arm (NIEA), and the Oakwood-Northwest-Wayne County Interceptor (ONWI) (Figure 1), serving the City of Detroit as well as the three largest Michigan counties by population: Wayne, Oakland, and Macomb. Composite samples collected over 24-h were used to compare the polyethylene glycol (PEG) precipitation and filtration methods, however, the larger volumes required by the virus adsorption-elution (VIRADEL) method necessitated a targeted approach with samples collected between 15:30 to 18:00 each afternoon. The samples were collected from the three interceptors at the point of discharge into the WRRF and maintained chilled on ice during transport to the lab for primary concentration and sample analysis.

### 2.1. Virus adsorption-elution method

**Figure 2:**
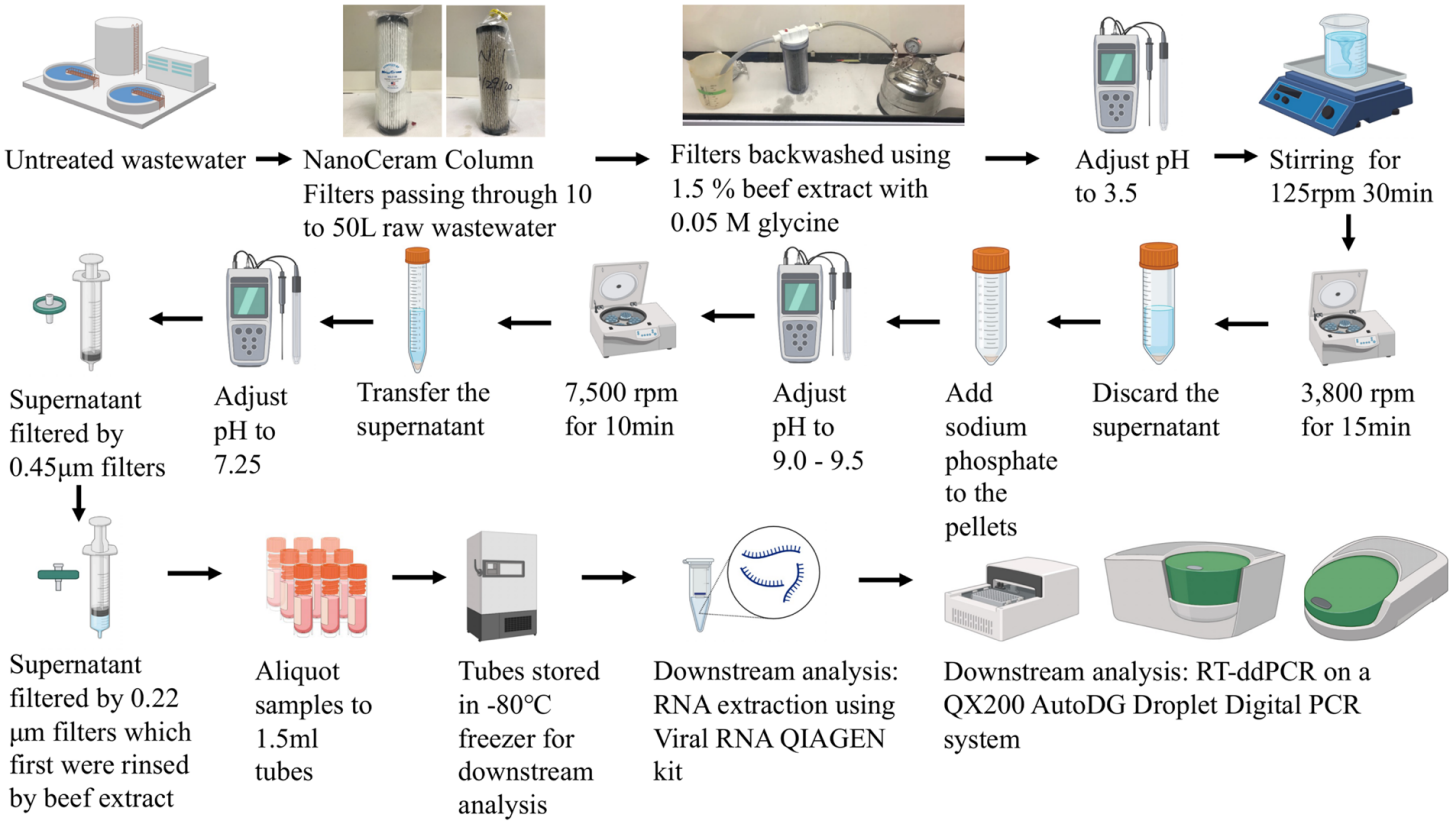
Illustrative flowchart of the VIRADEL concentration method and downstream analysis.

The United States Environmental Protection Agency virus adsorption-elution (VIRADEL) method employing electropositive or electronegative filters was reported to recover and concentrate viruses from wastewater samples previously (1–4, 13, 20, 35). Electronegative filters require preconditioning such as adjusting the pH, prior to downstream concentration processes. Electropositive filters do not require any preconditioning (20, 35). In this study, depending on the quantity of suspended solids in the wastewater, 10 to 50 L of untreated wastewater (grab sample) was passed through NanoCeram electropositive cartridge filters (Argonide, Sanford, FL, United States) at a rate less than 11 L/min using a previously described method (1–4). Flow meter readings were tracked at the beginning and end of each sampling event to measure the total volume of wastewater passing through the filters. Following sampling, the NanoCeram filters were transported on ice to the lab for sample analysis within 24 h. The elution process releases viral particles captured by the filters (20). Viruses were eluted using 1.5% beef extract containing 0.05 M glycine, based on a previously described method (1–4). Subsequently, the eluates containing viruses were flocculated by adjusting the pH, following multiple centrifugations and resuspension of particles in sodium phosphate. Afterwards, supernatants containing viruses were separated by adjusting the pH and centrifugation. Finally, the supernatants containing viruses were passed through 0.45 μm and 0.22 μm Millipore filters (MilliporeSigma, Burlington, MA, United States), which were followed by aliquoting and storage of the final aliquots at -80°C for downstream molecular analysis (1–4, 20). Bacteriophage Phi6 was applied as a proxy virus to estimate the recovery efficiency during virus concentration (3, 29, 39). Figure 2 demonstrates the workflow of the VIRADEL method.

### 2.2. Polyethylene glycol precipitation method

**Figure 3:**
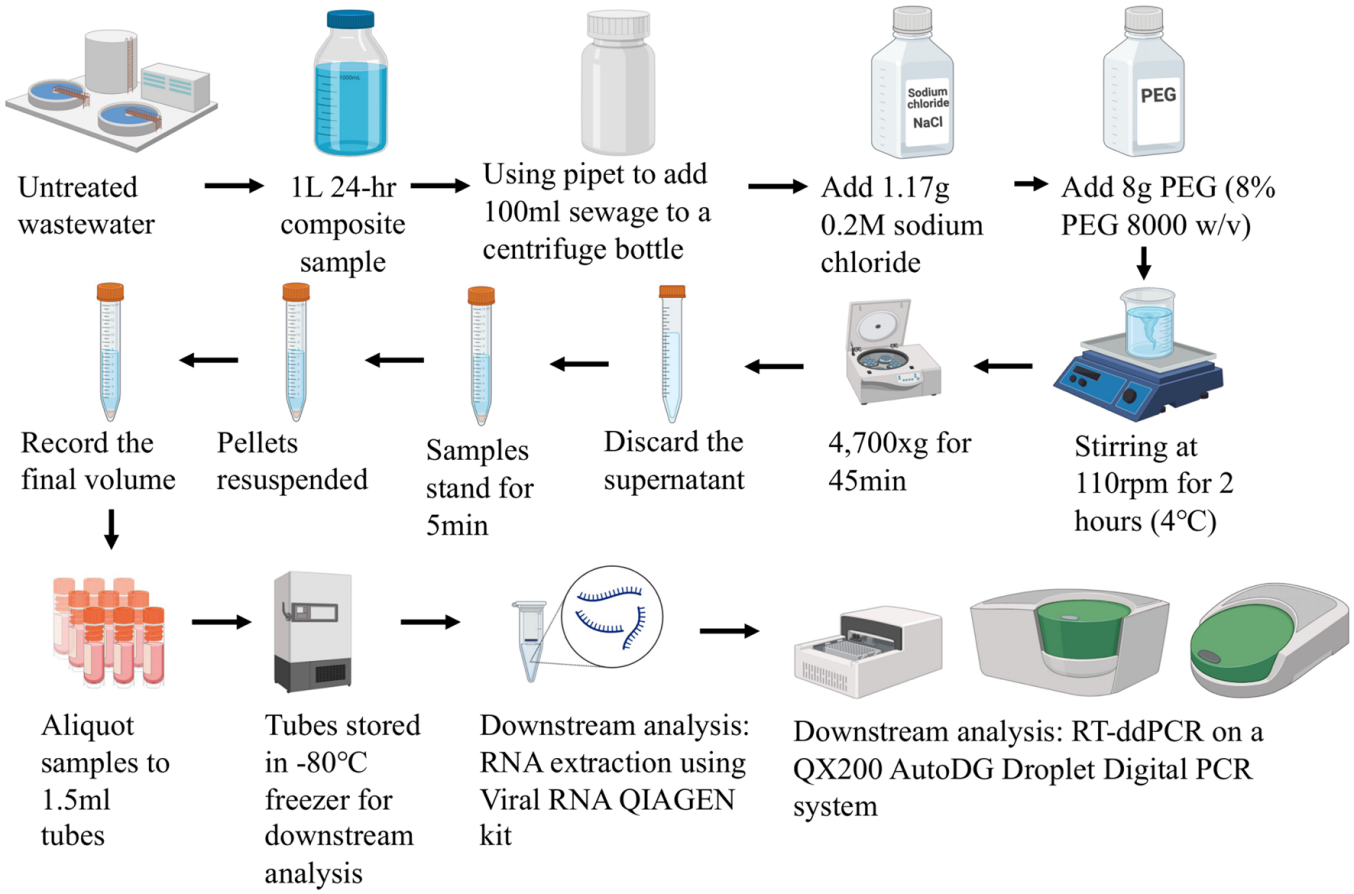
Illustrative flowchart of the PEG concentration method and downstream analysis.

From a 24-h composite sample of untreated wastewater collected in a 1 L Nalgene bottle, 100 mL samples were mixed with 0.2 M sodium chloride and 8% polyethylene glycol (w/v). Samples were mixed gently on a magnetic stirrer at 4°C for 2 h, followed by centrifugation at 4700 × *g* for 45 min at 4°C. The supernatant was removed, and the pellet was resuspended in the remaining liquid (approximately 2-3 mL). The final concentrate volumes were between 1 to 6 mL. All sample concentrates were then subjected to downstream analysis including RNA extraction and RT-ddPCR (Figure 3).

### 2.3. Filtration method

**Figure 4:**
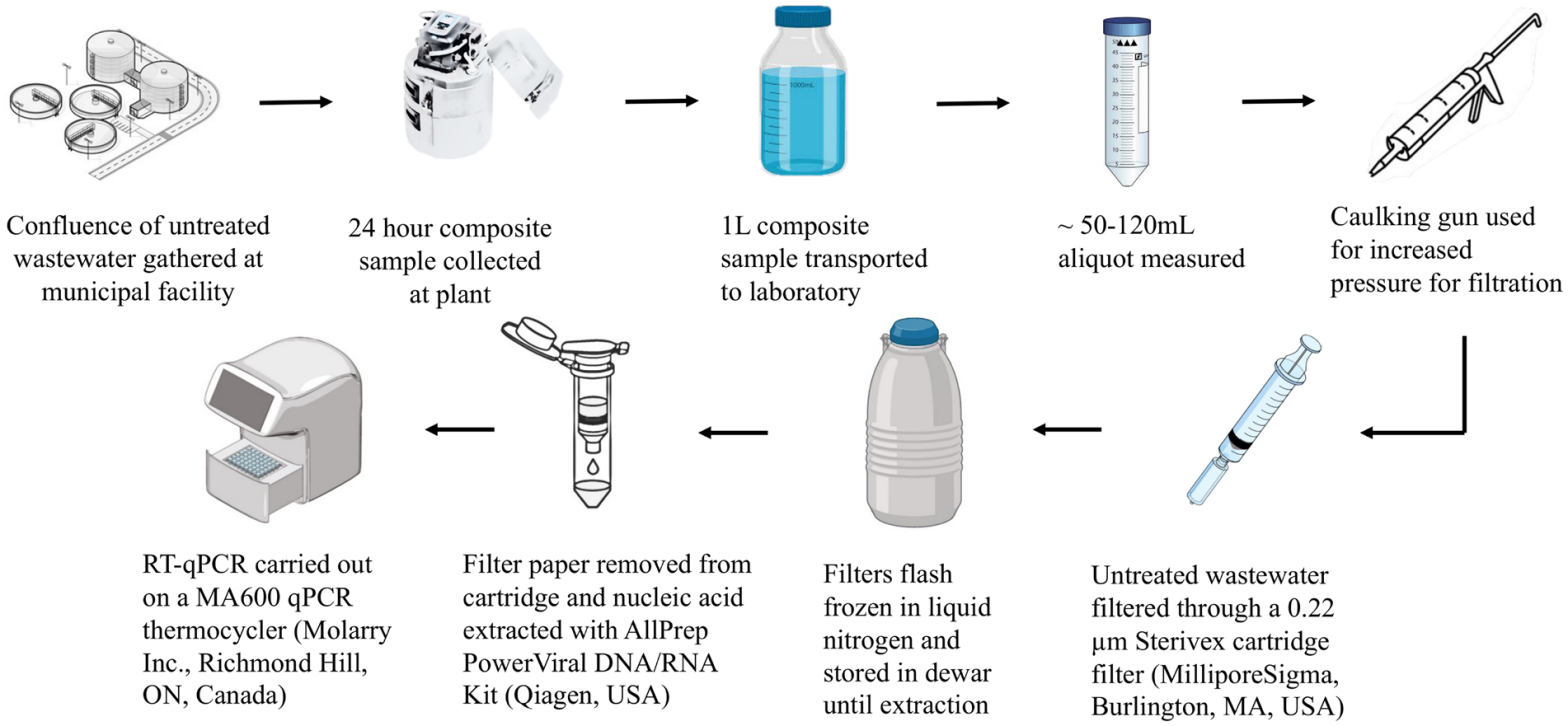
Illustrative flowchart of the filtration concentration method and downstream analysis.

Composite samples of raw wastewater collected as for the PEG method were concentrated by filtering 50-120 mL through 0.22 μm Sterivex PES cartridge filters (MilliporeSigma, Burlington, MA, United States) using a 50 mL syringe fitted into a caulking gun. Immediately following filtration, the filters were sealed and flash-frozen through immersion in liquid nitrogen as described previously (38). Subsequently, filters were subjected to downstream processes including RNA extraction and RT-qPCR (Figure 4).

### 2.4. RNA extraction, RT-ddPCR, RT-qPCR

Following VIRADEL and PEG methods, viral RNA was extracted using the QIAamp Viral RNA kit (Qiagen, Germantown, MD, United States), following the manufacturer’s protocol modified by use of 140 μL elution buffer to extract the viral RNA (1–4). RT-ddPCR was performed on a QX200 AutoDG Droplet Digital PCR system (Bio-Rad, Hercules, CA, United States), using the One-step RT-ddPCR Advanced Kit for Probes (Bio-Rad, Hercules, CA, United States) as described previously (2, 3). United States Centers for Disease Control and Prevention (US CDC) primers and probes that target the N1 and N2 genes of SARS-CoV-2 were used (2, 3, 13). N1 N2 gene Duplex Assay Reaction Mixture was reported previously (2, 3, 13). Following the preparation of the Duplex Mixture and oil droplets generation, samples were run on a C1000 Touch Thermal Cycler (Bio-Rad, Hercules, CA, United States) using the thermocycling conditions which were reported previously (2, 3, 13). Subsequently, the measurement of fluorescence was performed on a QX200 Droplet Reader (Bio-Rad, Hercules, CA, United States). For each RT-ddPCR run, positive controls (PTCs), negative controls (NTCs), and process negative controls were included, which were described previously (3). All samples were run in triplicate. The Limit of Detection (LOD) and Limit of Blank (LOB) for RT-ddPCR were described and determined previously (2, 3, 13).

Following the filtration method, filters were thawed, and RNA was extracted from the filters using the AllPrep PowerViral DNA/RNA kit (Qiagen, Germantown, MD, United States) modified by addition of 5% 2-mercaptoethanol (v/v). RNA was eluted in 50 μL of RNAse free water. Samples were not treated with DNase upon extraction. Assays for SARS-CoV-2 targeted regions of the nucleocapsid (N) gene using US CDC primers and probes for the N1 and N2 regions (40). Reagents were supplied by Integrated DNA Technologies (Coralville, IA, United States). Reactions contained 5 μL of RNA template mixed with 10 μL of 2 × RT-qPCR master mix (Takyon TM Dry One-Step RT Probe MasterMix No Rox, Eurogentec, Liège, Belgium) and primers and probes in a final reaction volume of 20 μL. Reaction inhibition was assessed using VetMAX XENO Internal Positive Control RNA (Applied Biosystems Corp., Waltham, MA, United States). Due to repeated incidence of inhibition with wastewater samples processed by filtration, template was diluted 1:5 in all reactions. Technical triplicates were run for detection of gene targets. Thermal cycling was performed using a MA6000 qPCR thermocycler (Sansure Biotech, Changsha, China). RT was performed at 48°C for 10 min, followed by polymerase activation at 95°C for 3 min, and 50 cycles of denaturation, annealing/extension at 95°C for 10 s, then 60°C for 45 s, respectively. The EDX SARS-CoV-2 synthetic RNA standard (Exact Diagnostics, Fort Worth, TX, United States) was used to create a 7-point standard curve to quantify N1 and N2 gene targets. No template controls yielded no amplification, and we report a limit of detection of 5 gene copies of N1 and N2 per reaction containing 5 μL of template RNA for RT-qPCR.

### 2.5. COVID-19 clinical data

**Figure 5:**
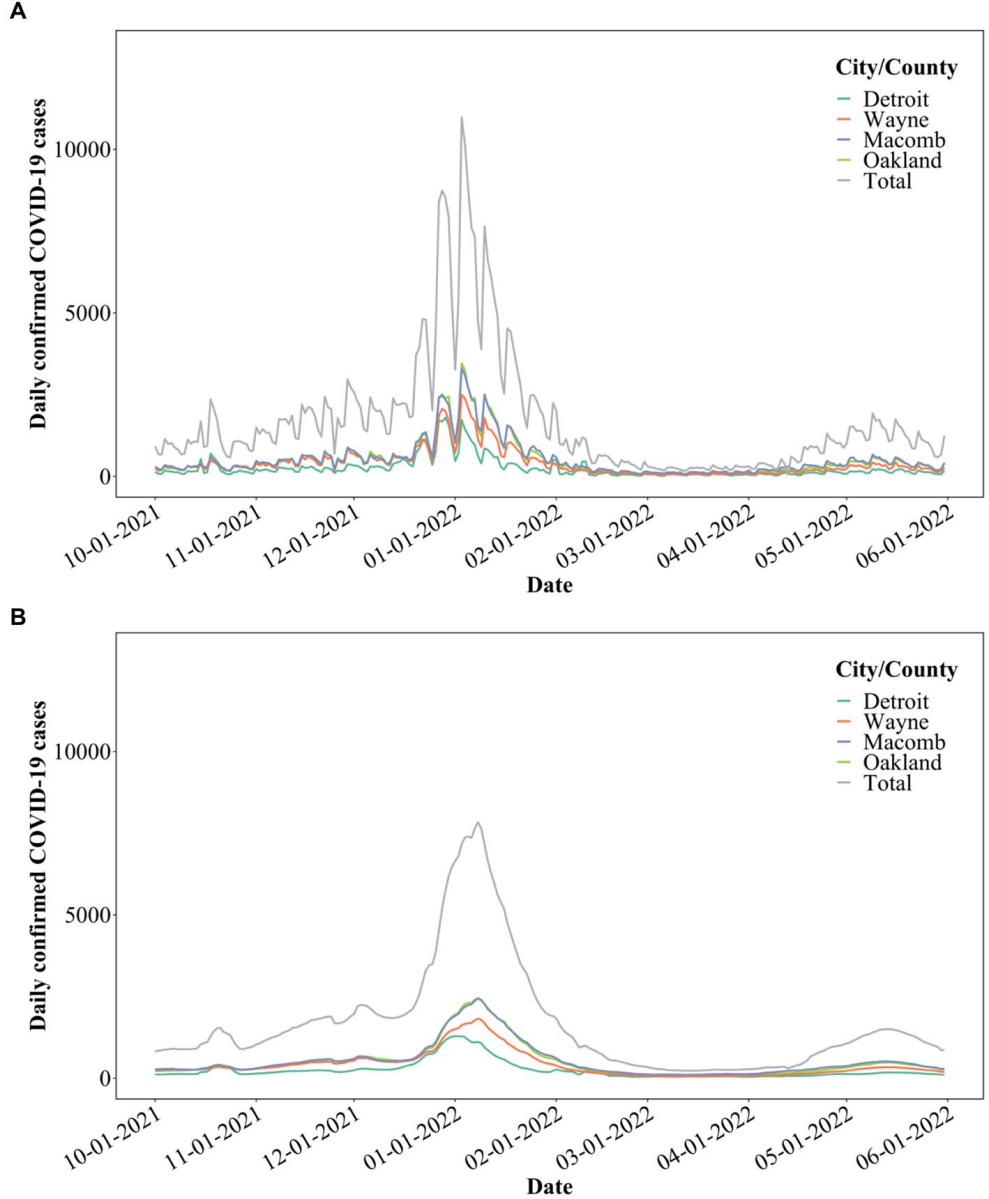
**(A)** COVID-19 cases in the City of Detroit, as well as Wayne, Macomb, and Oakland counties; **(B)** 7-day moving average of the COVID-19 cases.

Publicly available clinical data were accessed on August 22, 2022, for the period between October 1, 2021, and May 31, 2022, for the city of Detroit, as well as Wayne, Macomb, and Oakland counties (Figure 5A).^1^ Clinical data with a 7-day moving average (3, 41, 42) was used for further statistical analysis (Figure 5B). COVID-19 clinical data were only available per city/county for the Detroit metropolitan area. Each interceptor received wastewater from portions of each city/county. Therefore, only the total SARS-CoV-2 concentrations can be correlated to the total COVID-19 cases of each city/county (3, 13).

### 2.6. Data analysis and visualization

Data were tracked and organized using Microsoft Excel version 16.66.1. R version 4.1.3 was applied to perform data analysis including Pearson and Spearman correlations, Dynamic Time Warping (DTW), Time Lagged Cross Correlation (TLCC) and peak synchrony, depending primarily on ggplot2 package for visualization, and packages including dtw, synchrony, dplyr, and ggpubr. Missing data from samples were filled using linear interpolation for further analysis (3, 13, 43). For VIRADEL samples, 128 genes concentrations were measured for both N1 and N2 genes between 10/1/21 and 5/31/22. For PEG samples, 88 gene concentrations were measured for both N1 and N2 genes between 10/1/21 and 5/31/22. For filtration samples, 66 gene concentrations were measured for both N1 and N2 genes between 10/1/21 and 5/31/22. To perform correlation analysis between weekly gene concentrations and daily clinical cases, linear interpolation was conducted to generate daily data based on weekly measurements. The number of interpolated daily gene concentrations were 179, 199, and 210 for VIRADEL, PEG, and filtration, respectively.

To account for the changing flow in wastewater, dilution events, and variability in the solids portion of the wastewater, four approaches (flow rate, flow rate/population, TSS, flow rate×TSS) of normalizing the N1 and N2 gene concentrations (gc/L) were implemented using Eq. (1), Eq. (2), Eq. (3), and Eq. (4) (3, 44, 45). TSS, or “Total Suspended Solids,” is an estimate of the entire solids in wastewater in contrast to the liquid fraction or dissolved matter (45). In addition, other parameters, including sanitary percentage and Biological Oxygen Demand (BOD), proved ineffective for normalizing N1 and N2 gene concentrations for the Detroit area and other areas, thus, they were not considered in the current study (3, 9). SARS-CoV-2 gene concentrations measured in the wastewater following VIRADEL, PEG, and filtration methods are reported as gene copies per L (gc/L). The units after normalization using flow rate, flow rate/population, TSS, and flow rate×TSS, are gene copies per day (gc/day), gene copies per day per person (gc/day/person), gene copies per mg TSS (gc/mg TSS), and gene copies per L per pounds/day {gc/[L(pounds/day)]}, respectively.


(1) $$C_{\left(1\right)}=C_{N1\;or\;N2\;gene}*V*f$$

(2) $$C_{\left(2\right)}=\frac{C_{\left(1\right)}}{P}$$

(3) $$C_{\left(3\right)}=\frac{C_{N1\;or\;N2\;gene}}{TSS}$$

(4) $$C_{\left(4\right)}=\frac{C_{N1\;or\;N2\;gene}}{V*f*k*TSS}$$

$C_{\left(1\right)}$ is the normalized concentration of SARS-CoV-2 in gc/day. $C_{\left(2\right)}$ is the normalized concentration of SARS-CoV-2 in gc/day/person. $C_{\left(3\right)}$ is the normalized concentration of SARS-CoV-2 in gc/mg TSS. $C_{\left(4\right)}$ is the normalized concentration of SARS-CoV-2 in gc/[L(pounds/day)]. V is the volume of wastewater flowing into WWTP interceptors during sampling events (MGD). f is the conversion factor between L and MGD. k is the conversion factor between mg and pounds. P is the total population in the Detroit metropolitan area served by WRRF’s interceptors including ONWI, NIEA, and DRI. TSS represents the total suspended solids (mg/L).

#### 2.6.1. Correlations among N1 and N2 gene concentrations by VIRADEL, PEG, and filtration

Multiple studies investigated the applications of both Pearson and Spearman correlations on analyzing the relationship between wastewater viral concentrations of SARS-CoV-2 and COVID-19 clinical cases as well as the relationship among wastewater viral concentrations by different genes or methods (9, 46, 47). In this study, Pearson and Spearman correlations were performed among N1 and N2 gene concentrations {gc/L, gc/day, gc/day/person, gc/mg TSS, gc/(L[pounds/day)]} by VIRADEL, PEG, and filtration methods. The Pearson correlation measures how two time series among VIRADEL, PEG, and filtration gene concentrations covary during the study period, and indicate their linear relationships. The Spearman correlation coefficient is a simple and straightforward approach to analyze the degree of associations between two time series (48).

#### 2.6.2. Dynamic time warping

One commonly used algorithm for quantifying the similarities/dissimilarities between time series data is the Euclidean distance (ED), but numerous studies demonstrated that ED is insensitive to time shifting and patterns between time series since it compares the data points of time series in a settled sequence and cannot consider time shifting or patterns (49, 50). Dynamic time warping (DTW) is a well-established algorithm that circumvents the limitations of ED and compares two time series by computing dynamic distances between them considering regional distortions, time shifting, and the optimal warping that best aligns the time series between each other (50, 51). Therefore, similar patterns that occur at different times between time series can be considered as matching, thus, the similarity of time series can be evaluated considering their time shifting and shapes by DTW algorithm (50). The DTW algorithm was proposed previously (51).

The outcome of DTW analysis indicates two time series with the most similar patterns by calculating the minimum overall dissimilarity or the DTW minimum distance where data points on one time series best align data points on another time series (51). Multiple studies investigated the similarities between time series using DTW algorithm (50, 52, 53). However, to our knowledge, this is the first study to apply DTW algorithm to compare the similarities between wastewater gene concentrations data by three concentration methods (VIRADEL, PEG, and filtration), as well as comparing the similarities between wastewater gene concentrations data and COVID-19 clinical data. In this study, package dtw and related packages in R (version 4.1.3) were implemented to calculate DTW for the normalized {gc/day, gc/day/person, gc/mg TSS, and gc/[L(pounds/day)]} and non-normalized (gc/L) data to analyze the similarities/dissimilarities between VIRADEL, PEG, and filtration methods.

One limitation is that the minimum DTW distance can be affected by the scaling factor of time series data. For instance, the minimum DTW distance between PEG (gc/day/person) and COVID-19 cases can be smaller than the distance between VIRADEL (gc/day/person) and cases, indicating that PEG presents higher similarity to cases than VIRADEL. However, this was affected by the population factor which is a constant number but is not dynamic time series data. Using flow/population normalization including a constant factor intentionally changed the similarities among time series data. Therefore, the minimum DTW distance with flow/population normalized data was not considered for further discussions.

#### 2.6.3. Time lagged cross correlation and peak synchrony

To estimate the leading or lagging relationships between wastewater viral concentrations by three concentration methods (VIRADEL, PEG, and filtration) and total COVID-19 cases, TLCC and peak synchrony were performed where the total COVID-19 cases were shifted over time and correlated with wastewater viral concentrations for each concentration method. TLCC refers to correlations between two time series shifted relatively in time. It can identify the direction and relationship between two time series, for instance, a leader-follower relationship, where the leader time series develop a pattern which is repeated by the follower time series (54). TLCC is widely applied in analyzing time series especially delay, lead/lag time, and lagged cross correlation and so forth (44, 54–56). TLCC is an effective approach to estimate the dynamic relationships between two time series and demonstrate how they shift over time (44).

In this study, TLCC is measured by gradually shifting total COVID-19 cases between -20 days (lagging) and + 20 days (leading), and constantly calculating the Pearson’s correlation coefficients between two time series for each shifting. Peak synchrony occurs when the peak correlation is observed. For instance, if the peak correlation is observed at the center where the lag time or offset is 0 day, this condition indicates that the time series are most synchronized at day 0 demonstrating no shifting or lag time. However, the peak correlation can be at a different offset if one time series is leading or lagging another one. R package “synchrony,” “devtools,” and related packages were implemented to calculate the TLCC and peak synchrony between gene concentrations (both normalized and non-normalized data, by VIRADEL, PEG, and filtration methods) and 7-day moving average total COVID-19 cases.

## 3. Results

### 3.1. SARS-CoV-2 viral RNA concentrations in wastewater derived by three concentration methods spanning the transition between delta and omicron VOCs

RT-ddPCR (VIRADEL and PEG samples) and RT-qPCR (filtration samples) targeting the N1 and N2 genes was used to quantify SARS-CoV-2 RNA concentrations in wastewater samples collected at GLWA’s WRRF over 34 weeks. The study period captured the third major resurgence of COVID-19 cases in the region corresponding to the transition from SARS-CoV-2 Delta (B.1.617.2) variant to Omicron (B.1.1.529) variant (3, 44).

**Figure 6:**
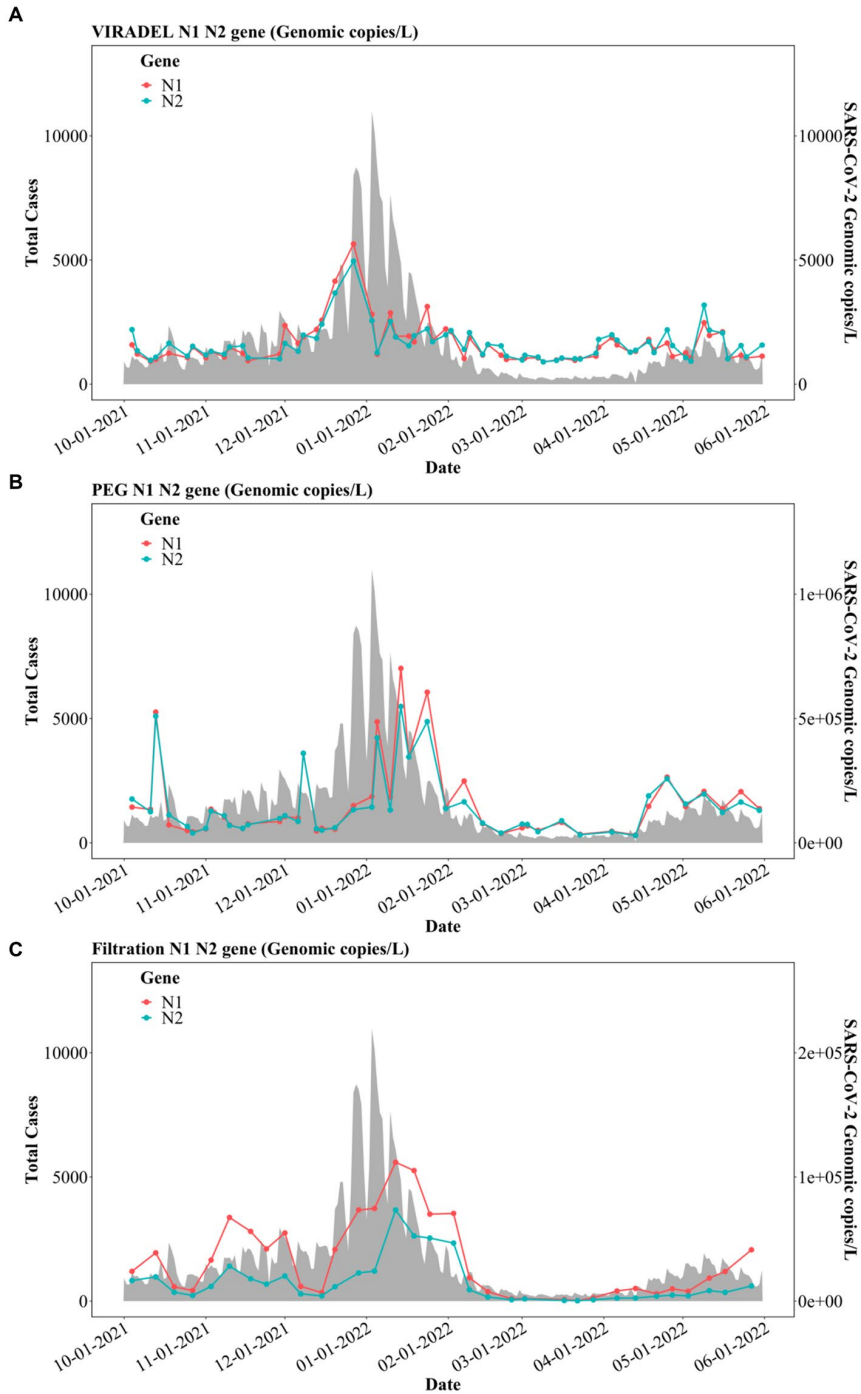
N1 and N2 gene concentrations (gc/L) by three concentration methods: **(A)** VIRADEL, **(B)** PEG, **(C)** Filtration, plotted against total COVID-19 cases.

Filtered samples yielded N1 and N2 gene concentrations higher than those of VIRADEL but lower than those of PEG, for both normalized and non-normalized data (Table 1). Filtered samples yielded mean N1 and N2 gene concentrations of 3.22E+04 and 1.50E+04 gc/L, respectively. VIRADEL samples yielded mean N1 and N2 gene concentrations of 1.61E+03 and 1.63E+03 gc/L, respectively. PEG samples yielded mean N1 and N2 gene concentrations of 1.61E+05 and 1.50E+05 gc/L, respectively. The overall observed trends of the VIRADEL total N1 and N2 gene concentrations increased steeply from early December 2021 and reached a peak in late December 2021 (Figure 6A), which heralded the major wave of COVID-19 cases in late December 2021 and early January 2022. Likewise, VIRADEL N1 and N2 gene concentrations increased in early April 2022, which preceded a resurgence of COVID-19 cases later in mid-April 2022.

**Table 1:** Total N1 and N2 gene concentrations measured in wastewater samples by VIRADEL, PEG, and filtration methods.

Gene	Methods			
		VIRADEL	PEG	Filtration
N1 (gc/L)	Maximum	5.64E+03	7.02E+05	1.12E+05
	Minimum	9.01E+02	3.18E+04	5.12E+02
	Mean	1.61E+03	1.61E+05	3.22E+04
N2 (gc/L)	Maximum	4.95E+03	5.48E+05	7.34E+04
	Minimum	9.01E+02	2.97E+04	3.13E+02
	Mean	1.63E+03	1.50E+05	1.50E+04
N1 (gc/day)	Maximum	5.24E+12	4.07E+14	7.40E+13
	Minimum	5.39E+11	2.36E+13	4.12E+11
	Mean	1.35E+12	1.18E+14	2.52E+13
N2 (gc/day)	Maximum	4.62E+12	3.18E+14	4.77E+13
	Minimum	5.84E+11	2.21E+13	2.93E+11
	Mean	1.37E+12	1.12E+14	1.14E+13
N1 (gc/day/person)	Maximum	1.69E+00	1.31E+02	2.38E+01
	Minimum	1.74E-01	7.58E+00	1.32E-01
	Mean	4.34E-01	3.79E+01	8.11E+00
N2 (gc/day/person)	Maximum	1.49E+00	1.02E+02	1.54E+01
	Minimum	1.88E-01	7.12E+00	9.43E-02
	Mean	4.41E-01	3.59E+01	3.65E+00
N1 (gc/mg TSS)	Maximum	5.82E+01	5.72E+03	1.19E+03
	Minimum	6.85E+00	2.20E+02	2.91E+00
	Mean	1.69E+01	1.53E+03	2.97E+02
N2 (gc/mg TSS)	Maximum	5.21E+01	4.66E+03	6.60E+02
	Minimum	6.71E+00	1.99E+02	3.84E+00
	Mean	1.69E+01	1.44E+03	1.41E+02
N1 (gc/(L(pounds/day)))	Maximum	2.94E-02	4.96E+00	8.83E-01
	Minimum	2.00E-03	6.48E-02	1.77E-03
	Mean	9.83E-03	1.01E+00	1.89E-01
N2 (gc/(L(pounds/day)))	Maximum	2.70E-02	4.01E+00	4.84E-01
	Minimum	1.96E-03	5.90E-02	2.06E-03
	Mean	9.84E-03	9.37E-01	9.27E-02

Previous reports have demonstrated that the VIRADEL method can serve as a leading indicator of COVID-19 cases (1, 3, 13). By contrast, PEG measured N1 and N2 gene concentrations were more variable and increased significantly in January 2022, lagging the major wave of COVID-19 infections (Figure 6B). PEG N1 and N2 gene concentrations increased simultaneously with the surge of COVID-19 cases in mid-April 2022, into May 2022. N1 and N2 gene concentrations yielded by the filtration approach increased in early November 2021 and decreased in early December 2021. Thereafter, gene concentrations rapidly increased starting in mid-December 2021, peaking in mid-January 2022, which later significantly decreased to a low level in February 2022 (Figure 6C). Notably, the peak in SARS-CoV-2 measured in wastewater by this approach was staggered, lagging the major wave of COVID-19 cases.

### 3.2. Correlations and similarity analysis among three concentration methods

#### 3.2.1. Correlations of N1 and N2 gene concentrations among three concentration methods

Multiple studies have applied Pearson and Spearman correlations to analyze the relationships between wastewater SARS-CoV-2 gene concentrations and COVID-19 cases (3, 9, 46), as well as the relationships among gene concentrations of SARS-CoV-2 in wastewater (47, 57). In this study, we tested the Pearson and Spearman correlations among N1 and N2 gene concentrations by VIRADEL, PEG, and filtration with normalized and non-normalized data (Table 2). A value of p that is less than 0.05 is considered statistically significant. For the non-normalized data (gc/L), the highest correlation was observed between PEG and filtration with N2 gene concentration (Pearson’s *r* = 0.67, Spearman’s *r* = 0.6). The lowest correlation was found between VIRADEL and PEG for N2 gene concentration (Pearson’s *r* = 0.12, Spearman’s *r* = 0.34). For non-normalized data (gc/L), the correlations between PEG and filtration were stronger than the correlations between VIRADEL and filtration, which in turn was stronger than the correlations between VIRADEL and PEG. For normalized data, the highest correlation was found between PEG and filtration for N1 (Pearson’s *r* = 0.73, Spearman’s *r* = 0.66) and N2 (Pearson’s *r* = 0.76, Spearman’s *r* = 0.64) gene concentrations in gc/[L(pounds/day)]. Significant correlations (Pearson coefficient > 0.63, Spearman coefficient > 0.6) were observed between PEG and filtration in gc/L, gc/mg TSS and gc/(L(pounds/day)) (Table 2). VIRADEL has stronger correlation to filtration than to PEG for both normalized and non-normalized data.

**Table 2:** Correlation coefficients among gene concentrations by VIRADEL, PEG, and filtration methods.

Methods (Unit)	Gene (Correlation)			
	N1 (Pearson)	N1 (Spearman)	N2 (Pearson)	N2 (Spearman)
V-P (gc/L)	0.17	0.36	0.12	0.34
V-P (gc/day)	0.10	0.17	0.11	0.13
V-P (gc/day/person)	0.10	0.17	0.11	0.13
V-P (gc/mg TSS)	0.29	0.41	0.27	0.46
V-P (gc/(L(pounds/day)))	0.46	0.58	0.43	0.62
V-F (gc/L)	0.41	0.46	0.23	0.40
V-F (gc/day)	0.26	0.13	0.04	0.05
V-F (gc/day/person)	0.26	0.13	0.04	0.05
V-F (gc/mg TSS)	0.49	0.47	0.27	0.39
V-F (gc/(L(pounds/day)))	0.59	0.64	0.41	0.60
P-F (gc/L)	0.63	0.60	0.67	0.60
P-F (gc/day)	0.46	0.51	0.45	0.50
P-F (gc/day/person)	0.46	0.51	0.45	0.50
P-F (gc/mg TSS)	0.67	0.63	0.68	0.60
P-F (gc/(L(pounds/day)))	0.73	0.66	0.76	0.64

V represents VIRADEL, P represents PEG, F represents filtration.

Normalizations using flow rate and flow rate/population reduced the correlations of gene concentrations among VIRADEL, PEG, and filtration compared to the correlations using the non-normalized data (gc/L) (Table 2). For instance, both Pearson and Spearman correlation coefficients between PEG and filtration were reduced from 0.67 (N2, Pearson, gc/L) and 0.6 (N2, Spearman, gc/L) to 0.45 (N2, Pearson, gc/day) and 0.5 (N2, Spearman, gc/day), respectively (Table 2). Conversely, normalizations using TSS and flow rate×TSS enhanced the correlations of gene concentrations among the three methods. For instance, higher correlation coefficients (Pearson’s r ranged from 0.73 (N1 gene) to 0.76 (N2 gene), Spearman’s r ranged from 0.64 (N2 gene) to 0.66 (N1 gene), all *p* < 0.05) were observed between PEG and filtration gene concentrations after normalization using flow rate×TSS compared to the correlation coefficients for non-normalized data (gc/L) (Pearson’s r ranged from 0.63 (N1 gene) to 0.67 (N2 gene), Spearman’s *r* = 0.6 (both N1 and N2 gene), all *p* < 0.05).

#### 3.2.2. Dynamic time warping of N1 and N2 gene concentrations among three concentration methods

Detecting patterns and comparing similarities of gene concentration time series data are critical for comparing the concentration methods. Dynamic time warping (DTW) identifies the most similar patterns and the optimal warping match between two time series by calculating the minimum DTW distance (51, 53, 58). Shorter DTW distances indicate higher degree of similarity in patterns/shapes between two time series (59, 60). Table 3 presents the DTW minimum distances among the N1 and N2 gene concentrations by VIRADEL, PEG, and filtration methods. Smallest DTW distances were observed between VIRADEL and filtration for both non-normalized and normalized data, which indicated that VIRADEL has a higher degree of similarity with filtration than with PEG. Largest DTW distances were observed between VIRADEL and PEG for both non-normalized and normalized data, indicating that VIRADEL and PEG have the least similarity. This finding was consistent with the sampling and concentration mechanisms since VIRADEL targets free and suspended viral particles in the dissolved phase of wastewater, whereas PEG targets particle-associated viruses, some of which may represent previously shed and accumulated viruses in the sewer stream (3, 22).

**Table 3:** Dynamic time warping (DTW) minimum distances among gene concentrations by VIRADEL, PEG, and filtration methods.

Methods (Unit)	Gene	
	N1	N2
V-P (gc/L)	4.37E+07	4.07E+07
V-P (gc/day)	3.23E+16	3.06E+16
V-P (gc/day/person)	1.04E+04	9.83E+03
V-P (gc/mg TSS)	3.93E+05	3.68E+05
V-P (gc/(L(pounds/day)))	2.42E+02	2.23E+02
V-F (gc/L)	7.51E+06	3.14E+06
V-F (gc/day)	5.87E+15	2.35E+15
V-F (gc/day/person)	1.89E+03	7.56E+02
V-F (gc/mg TSS)	6.74E+04	2.84E+04
V-F (gc/(L(pounds/day)))	4.33E+01	1.92E+01
P-F (gc/L)	2.60E+07	2.85E+07
P-F (gc/day)	1.83E+16	2.27E+16
P-F (gc/day/person)	5.89E+03	7.30E+03
P-F (gc/mg TSS)	2.45E+05	2.94E+05
P-F (gc/(L(pounds/day)))	1.46E+02	1.66E+02

V represents VIRADEL, P represents PEG, F represents filtration.

Normalization using flow rate decreased the similarity among methods. For instance, the DTW distance between VIRADEL and filtration increased significantly after normalizing using flow rate (gc/day), indicating that the similarity between VIRADEL and filtration was reduced after normalization (Table 3). Conversely, normalization using TSS and flow rate×TSS strengthened the similarity among methods. For instance, the DTW distances decreased in gc/mg TSS and gc/(L(pounds/day)) comparing to the DTW distance in gc/L among the methods, indicating the improvement of similarity among methods after normalization (Table 3).

### 3.3. Similarity and TLCC analysis between three concentration methods and COVID-19 cases

#### 3.3.1. Dynamic time warping between three concentration methods and COVID-19 cases

Wastewater surveillance data for COVID-19 primarily contain temporal data of viral gene concentrations and clinical cases. DTW analysis were performed between gene concentrations derived from the three concentration methods (VIRADEL, PEG, and filtration) and the 7-day moving average of total COVID-19 cases for both normalized and non-normalized data. For non-normalized data (gc/L), the smallest DTW distance was found between VIRADEL and total COVID-19 cases (Table 4). This indicates that VIRADEL (gc/L) has the highest similarity to total COVID-19 cases among the three concentration methods tested. The largest DTW distance was found between PEG (gc/L) and total COVID-19 cases, indicating the PEG method for concentration yields the least similarity to clinical cases. Normalizing gene concentration data using flow (gc/day) demonstrated similar findings. Conversely, normalization using TSS and flow×TSS can significantly increase the similarity between PEG and total COVID-19 cases but concurrently decrease the similarity between VIRADEL and total COVID-19 cases. Specifically, for normalized data (gc/mg TSS, gc/L(pounds/day)), the smallest DTW distance was identified between PEG and total COVID-19 cases, indicating the PEG has the highest similarity to total COVID-19 cases. The largest DTW distance was identified between VIRADEL and COVID-19 cases, indicating that VIRADEL has the lowest similarity to total COVID-19 cases.

**Table 4:** Dynamic time warping (DTW) minimum distances between gene concentrations by VIRADEL, PEG, as well as filtration methods and total COVID-19 cases.

Method-cases (Unit)	Gene	
	N1	N2
V-cases (gc/L)	1.04E+05	1.28E+05
V-cases (gc/day)	4.72E+14	4.86E+14
V-cases (gc/day/person)	4.61E+05	4.61E+05
V-cases (gc/mg TSS)	4.55E+05	4.54E+05
V-cases (gc/(L(pounds/day)))	4.61E+05	4.61E+05
P-cases (gc/L)	4.39E+07	4.08E+07
P-cases (gc/day)	3.30E+16	3.14E+16
P-cases (gc/day/person)	4.43E+05	4.42E+05
P-cases (gc/mg TSS)	9.87E+04	1.14E+05
P-cases (gc/(L(pounds/day)))	4.61E+05	4.61E+05
F-cases (gc/L)	7.35E+06	2.95E+06
F-cases (gc/day)	6.20E+15	2.82E+15
F-cases (gc/day/person)	4.57E+05	4.59E+05
F-cases (gc/mg TSS)	2.87E+05	3.92E+05
F-cases (gc/(L(pounds/day)))	4.61E+05	4.61E+05

V represents VIRADEL, P represents PEG, F represents filtration, cases represents total 7-day-moving-average clinical cases.

#### 3.3.2. Time lagged cross correlation and peak synchrony between three concentration methods and COVID-19 cases

The relative timing of the wastewater gene concentrations {gc/L, gc/day, gc/day/person, gc/mg TSS, and gc/[L(pounds/day)]} of VIRADEL, PEG and filtration were compared to the total COVID-19 cases using TLCC and peak synchrony. To evaluate if wastewater viral concentrations of the three methods lead or lag COVID-19 cases, the total COVID-19 case data were shifted by a period of −20 (lagging) to +20 days (leading) and the Pearson’s correlation coefficients were calculated between cases and wastewater viral gene concentration for each shift. The leading or lagging metric varied for each method, which was determined by comparing the strongest Pearson’s correlation coefficient.

**Figure 7:**
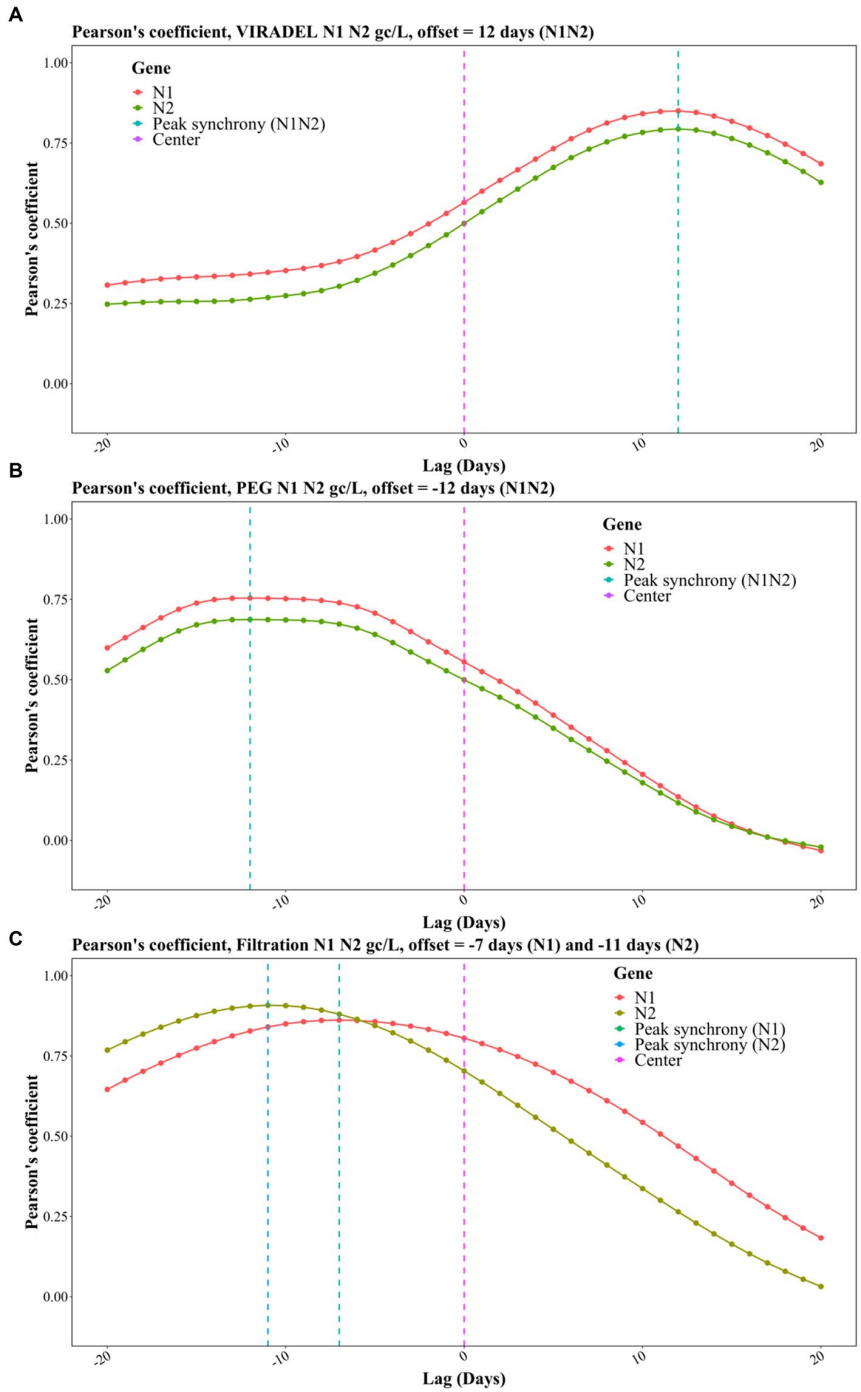
Pearson correlation coefficients for TLCC and peak synchrony between wastewater viral concentrations and COVID-19 cases with offsets between −20 (lagging) and + 20 (leading) days for the three methods, including **(A)** VIRADEL, **(B)** PEG, and **(C)** Filtration.

For the VIRADEL method, both N1 and N2 gene concentrations (gc/L) were strongly correlated with COVID-19 cases, covering shifting windows between −20 and + 20 days (Figure 7A). The highest correlation coefficient was observed when offset is +12 days (Figure 7A), indicating that SARS-CoV-2 gene concentrations (gc/L) in wastewater by the VIRADEL method lead COVID-19 cases by 12 days, which concurred with previous findings of a 35-day lead time of gene concentrations preceding total COVID-19 cases prior to the Omicron surge (3). For both non-normalized and normalized data, VIRADEL always led COVID-19 cases with a variety of lead times (Table 5).

**Table 5:** Lead/lag time between wastewater viral concentrations by VIRADEL, PEG, as well as filtration methods and total COVID-19 cases.

Units	Method (Gene)					
	V (N1)	V (N2)	P (N1)	P (N2)	F (N1)	F (N2)
gc/L*	+12	+12	-12	-12	-7	-11
gc/day	+13	+13	-6	-6	-2	-10
gc/day/person	+13	+13	-6	-6	-2	-10
gc/mg TSS	+11	+11	-9	-9	-7	-12
gc/(L(pounds/day))	+9	+9	-14	-14	-11	-13

V represents VIRADEL, P represents PEG, F represents filtration, * was demonstrated in Figure 7., + indicates lead time, – indicates lag time.

For the PEG method (gc/L), the strongest correlation coefficients were observed with an offset of -12 days, indicating that SARS-CoV-2 gene concentrations by the PEG method lagged reported COVID-19 cases by 12 days during the study period (Figure 7B).

For the filtration method (gc/L), the highest correlation coefficient was observed with an offset of -7 days for the N1 gene and -11 days for the N2 gene, indicating that SARS-CoV-2 gene concentrations in wastewater lagged reported COVID-19 cases for 7 days (N1) and 11 days (N2), respectively (Figure 7C). Likewise, similar observations were found for normalized data where the filtration method yielded data that lagged clinical cases (Table 5). Table 5 summarized the lead/lag time between VIRADEL, PEG, and filtration methods and total COVID-19 cases. The length of the leading or lagging time differed with dissimilar normalizations. However, the leading or lagging pattern of each method did not change, where VIRADEL measurements were always leading COVID-19 cases, whereas PEG and filtration measurements routinely lagged COVID-19 cases.

## 4. Discussion

There is an ongoing effort to optimize methods to recover and concentrate SARS-CoV-2 from wastewater in support of actionable public health outcomes (33, 61). In this study, three concentration methods were evaluated for concentrating SARS-CoV-2 from wastewater, spanning the transition between Delta and Omicron variants circulating in the Detroit, MI metropolitan area. The three methods share common characteristics, especially downstream where they follow similar procedures of nucleic acid extraction and quantification such as RT-ddPCR or RT-qPCR. Likewise, their recovery efficiencies are reported as comparable (2, 3, 22, 34, 62).

### 4.1. VIRADEL: opportunities and obstacles

Several studies have previously adopted VIRADEL as the concentration method for SARS-CoV-2 in wastewater (1–4, 13). An attribute of the VIRADEL method is the ability to process large volumes (10 – 50 L) of wastewater, thus facilitating capture of free and suspended viral particles that are arguably most representative of viruses shed by recently infected individuals (3, 35). This establishes VIRADEL as a concentration method capable to provide early warning that leads case reporting (1, 3), which was also verified by TLCC analysis in this study (Table 5). Limiting widescale adoption of VIRADEL is labor-intensive preparation of sampling units which require extensive washing and disinfection prior to use. VIRADEL (63) also requires access to large volumes of wastewater which may not be available to all researchers. Further, the required large volumes may necessitate use of grab samples which typically yield higher variability than composite samples which is the sampling method of choice for many wastewater surveillance efforts (64). VIRADEL requires trained personnel for comparatively laborious work with limited samples (*n* = 15) processed over a relatively long time (4-6 h). VIRADEL also requires multiple large centrifuges as well as expensive and at times, supply chain-limited consumables. Therefore, VIRADEL may not be an ideal choice for routine wastewater surveillance projects in common microbiology laboratories. However, it was clear from the comparative analysis conducted that VIRADEL has clear potential to be implemented as a tool to provide early warning to inform public health actions (1, 3, 13).

### 4.2. PEG: opportunities and obstacles

Apart from requiring access to a centrifuge, the consumables required are widely available and relatively inexpensive, lending itself as one of the most broadly applied concentration methods for routine wastewater surveillance (3, 22, 31, 33, 62, 63). On the other hand, PEG is restricted to processing smaller volumes of wastewater (usually 0.05 to 2 L) and only a portion of the sample pellet is used to recover and extract RNA, which can be affected by the variation of samples and representation of all viruses in wastewater (3, 22, 33, 35).

Unlike VIRADEL, PEG targets particle-associated viruses consistent with reports that identify solids as the phase yielding highest SARS-CoV-2 concentrations in wastewater (63). While a fraction of these particles will represent recently deposited SARS-CoV-2, the majority may represent previously shed and accumulated viruses in the sewer stream and later resuspended during flow fluctuations, thus providing a mechanism for the method to yield data lagging clinical COVID-19 cases. Though the exact mechanism of PEG is not well understood, several studies proposed that it captures viruses that are sorbed to larger precipitates and solids, consistent with a high quantity of TSS in wastewater (3, 22). In this study, through the DTW analysis, PEG yielded data were normalized using TSS and flow × TSS, which increased the degree of similarity between PEG and total COVID-19 cases (Table 4). This demonstrated that PEG yielded data were largely affected by the presence of TSS. VIRADEL, instead, captured free and suspended viruses in the supernatant wastewater. Thus, normalizing the VIRADEL data using TSS and flow × TSS decreased the similarity between VIRADEL and cases (Table 4).

Through the TLCC analysis, this study also demonstrated that PEG gene concentrations lagged COVID-19 cases (Table 5), which embraced the aforementioned sampling mechanism of PEG (22). PEG method did not provide an early warning (leading window) for COVID-19 cases which concurred with our previous findings, whereas VIRADEL provided early warnings ahead of clinical cases while PEG lagged clinical cases for the Detroit area (3).

However, several studies using PEG provided early warnings of impending COVID-19 cases (65). Notably, in these studies, PEG was applied to different types of samples such as primary sewage sludge, which is a different sample matrix from untreated wastewater samples, thus needing more investigation on the impact of sample types on early warnings (65). Kumar et al. (66) identified early warnings using PEG in the early stage of the pandemic in August 2020 in India (66). PEG and other concentration methods [such as ultrafiltration (17, 67) and adsorption-precipitation (68)] identified early warnings in the early stage of the pandemic when testing capacities were largely limited, and societal responses to the pandemic and clinical data reporting were significantly delayed (3, 69). In addition, earlier prevalent COVID-19 variants including Alpha, Beta and Gamma were reported with longer incubation times than Delta and Omicron variants, leading to prolonged early warning potentials of wastewater surveillance in the early stages of the pandemic (70).

Though PEG was reported to provide early warnings, it may have a shorter early warning window than VIRADEL due to the fundamental disparity of their targets, that being newly contributed free and suspended viral particles versus particle-attached virus, some of which may be considered previously shed and accumulated and subsequently resuspended from sediment (3). In the current study, PEG was shown to lag clinical cases while VIRADEL was leading clinical cases for both normalized and non-normalized data (Table 5). Overall, the early warning potential of PEG needs further investigations in terms of sample types, sampling mechanisms and locations, stage of the epidemic, among other factors.

### 4.3. Filtration: opportunities and obstacles

Filtration is commonly applied to recover and concentrate viral RNA in water samples. It achieves generally good recovery efficiencies, is relatively inexpensive using commonly available lab equipment and simple protocols and provides consistent performance and inclusive measurement since it captures viruses from both solids and liquid fractions by nature of forcing free viral particles across trapped solids (33, 37). However, filtration has several drawbacks. First, the number of available filtration units restricts the number of samples that can be processed concurrently (33). Meanwhile, clogging of filters can occur due to high variations of turbidity in wastewater. While this can be offset in part by use of a caulking gun to exert more pressure on the sample being filtered, in reality, volumes are limited to ~0.1 L. Additionally, filtration measurements lagged the COVID-19 clinical cases in the current study, thus, its ability to provide early warnings for impending cases is called into question. The recovery efficiencies also differ with different filters (33).

### 4.4. Future directions

The mechanism and implications of primarily collecting viruses attached to solids that may have settled and resuspended before sampling, such as by the PEG, needs further investigations. Notably, multiple studies have reported that the integrity of SARS-CoV-2 RNA was higher when sorbed to suspended solids, organic matter, and large bio-solids which provide protection from predation and inactivation. This can be explained by the hydrophobicity of SARS-CoV-2 viral particles, leading to their adherence to wastewater solids and longer persistence compared to free viruses in the supernatant wastewater (71–73).

The implications of seasonal variations in SARS-CoV-2 persistence in wastewater needs further investigations. Seasonal variations of wastewater temperature and pH are reported to affect the persistence of viral RNA in wastewater (74). However, SARS-CoV-2 RNA was shown to be highly stable at 4°C aqueous environment or in a wide pH range at room temperature (75, 76). Multiple studies reported the detectability and persistence of SARS-CoV-2 RNA in untreated wastewater solids samples. For instance, researchers found that SARS-CoV-2 RNA was consistently detected for 29 days and 64 days at 4°C and -20°C, respectively in wastewater solids pelleted by centrifugation (77). Another study indicated that only minimal reduction of SARS-CoV-2 RNA was observed for wastewater solids samples after 100 days (78). Additionally, researchers established models to indicate that viral RNA can be detected in wastewater even with long sewer travel time (100 h), especially with lower average wastewater temperature in northern cities such as Detroit (74). A recent study also indicated that biofilms could mediate the fate of SARS-CoV-2 in wastewater, especially leading the viral RNA to prolonged presence (79).

The effect of varying sampling volumes needs further investigation. Some studies indicated that a larger sampling volume can increase the sensitivity of the sampling method, suggesting that it will detect lower levels of viral RNA in wastewater samples (80). Similarly, researchers suggested that processing of larger sample volumes may help to lower the method detection limits (74). But at the same time, keeping the required samples sizes low can reduce shipping costs between sampling location and the analytical laboratory as well as reduce space for storage (74). Other researchers indicated that detection sensitivity can be improved by increasing the sample volume from 100 mL to 500 mL wastewater for testing SARS-CoV-2 (6).

However, other researchers presented that large-volume sampling did not significantly enhance the sensitivity of methods (81). For instance, Zheng et al. (81) found that wastewater concentration methods (they used ultracentrifugation) using less volume of wastewater was preferable than larger volume of wastewater in terms of sensitivity for testing SARS-CoV-2. The study revealed that when using the same concentration methods, no significant difference was observed in the viral RNA concentrations between experiments conducted with a larger volume of wastewater and those conducted with a smaller volume (81). Some studies indicated that a larger sampling volume may also dilute the wastewater sample, which can lead to a lower viral RNA concentration (82).

Overall, the sampling volume for wastewater surveillance of SARS-CoV-2 using different concentration methods will depend on several factors, including the sensitivity of the method, the concentration of viral RNA in the wastewater, and the size of the population being monitored. It is critical to consider and address these factors when analyzing wastewater surveillance data and more in-depth research on how the sampling volume affect statistical results are needed.

The time of sampling may potentially affect results in sewershed sampling. The effect of sampling time in large interceptors, like the ones sampled in this study, is less significant, since the interceptor wastewater is mixed at the pumping stations. A few studies have reported gene concentration varying on an hourly basis (83, 84) although the temporal variability of SARS-CoV-2 concentrations in wastewater remains ambiguous (83, 85). It has been suggested that composite samples may circumvent the within-day variation of viral concentrations (83). Whereas both the PEG and filtration methods used composite samples, the large volume required for VIRADEL necessitated separate sampling which was conducted over a period of several hours to help reduce temporal variability. Further, considering the vast sewersheds and population of nearly 4 million people that GLWA’s three interceptors serve, the concentrations of SARS-CoV-2 in wastewater may be highly diluted and within-day variations can be negligible. Future studies are called to examine within-day variation of SARS-CoV-2.

Admittedly, there are caveats to the current study that should be considered and discussed. The study period was limited to the transition between Delta and Omicron VOCs that occurred between fall 2021 and winter 2022. With each successive resurgence of COVID-19, differences are reported related to disease trajectory including incubation time, shedding dynamics and disease severity (18, 86). For instance, the incubation time was shorter during the Omicron surge compared to the previous variants, inevitably reducing the early warning potentials of wastewater surveillance in the later stage of the pandemic (3, 86). Further, the changing viral shedding dynamics, viral decay kinetics, and shedding duration of the Omicron variant are not well understood and many uncertainties remain (18, 87). As such, the lead and lag times reported here cannot be extrapolated to past or future SARS-CoV-2 variants. In addition, sampling frequency was limited to weekly samples and thus less informative for establishing time series or less likely to depict accurately the actual fluctuations of wastewater viral concentrations (cdc.gov). Feng et al. (88) proposed a minimum of two samples collected weekly to establish the time series data of wastewater viral concentrations for continuous trend analysis. Some researchers have even suggested daily or very frequent sampling, if the laboratory is capable of handling increased numbers of samples, considering rapid resurgence of COVID-19 cases (89). Indeed, the filtration method has been used to analyze samples 5 days weekly since the emergence of the Omicron VOC as part of Ontario’s Wastewater Surveillance Initiative in the Windsor-Essex region located across the international border with Detroit (Q. Geng, R. Corchis-Scott, R.M. McKay, unpublished). While SARS-CoV-2 signal intensity derived from this approach does not provide a clear early warning of clinical cases, preliminary analysis supports its use as a leading indicator of COVID-19-related hospitalizations in the region (Q. Geng, R. Corchis-Scott, R.M. McKay, unpublished). This is important considering that clinical testing capacity across North America was overwhelmed by infections attributed to Omicron and is thus no longer a reliable indicator of disease prevalence (90).

## 5. Conclusion

This study is among the first to implement, evaluate, and compare commonly applied wastewater virus concentration methodologies to recover and concentrate SARS-CoV-2 from wastewater amid the transition between Delta and Omicron VOCs. Analytical approaches, including Pearson and Spearman correlations, Dynamic Time Warping (DTW), and Time Lagged Cross Correlation (TLCC) and peak synchrony, were performed to analyze the relations among three methods as well as the relations between methods and COVID-19 cases. To our knowledge, this is the only study to implement Dynamic Time Warping to compare wastewater surveillance time series data and successfully identify the similarities/dissimilarities among the methods and between methods and clinical data. The analytical approach used can be applied to different sample processing and concentration methods under various pandemic scenarios to evaluate method efficacy for different public health practices.

## Data availability statement

The original contributions presented in the study are included in the article/supplementary material, further inquiries can be directed to the corresponding author.

## Author contributions

LZ: methodology, investigation, data acquisition, data curation, formal analysis, visualization, writing–original draft, and writing–review and editing. QG and RC-S: methodology, investigation, data acquisition, and writing–review and editing. RM: methodology, investigation, data acquisition, resources, supervision, funding acquisition, and writing–review and editing. JN: funding acquisition and writing–review and editing. IX: conceptualization, funding acquisition, methodology, investigation, project administration, resources, supervision, and writing–review and editing.

## Funding

This study was funded by the Michigan Department of Health and Human Services (MDHHS) and the Great Lakes Water Authority (GLWA). RMM acknowledges support from Ontario Genomics (COVID-19 Regional Genomics Initiative) with additional support provided by the Canada Foundation for Innovation–Exceptional Opportunities Fund (COVID-19 program) and National Institutes of Health [1P01ES028939-01], and National Science Foundation [OCE-1840715] awards to the Bowling Green State University Great Lakes Center for Fresh Waters and Human Health.

## Conflict of interest

The authors declare that the research was conducted in the absence of any commercial or financial relationships that could be construed as a potential conflict of interest.

## Publisher’s note

All claims expressed in this article are solely those of the authors and do not necessarily represent those of their affiliated organizations, or those of the publisher, the editors and the reviewers. Any product that may be evaluated in this article, or claim that may be made by its manufacturer, is not guaranteed or endorsed by the publisher.

## Footnotes

^1^ michigan.gov/coronavirus/stats
